# The relationship between maternal glucose concentrations, gestational diabetes mellitus, placental weight, and placental vascular malperfusion lesions: A retrospective study of a U.S. pregnancy cohort

**DOI:** 10.1371/journal.pone.0325415

**Published:** 2026-03-03

**Authors:** Amrita Arcot, Kelly Gallagher, Jeffery A. Goldstein, Alison D. Gernand

**Affiliations:** 1 Department of Nutritional Sciences, The Pennsylvania State University, University Park, Pennsylvania, United States of America; 2 Ross and Carol Nese College of Nursing, The Pennsylvania State University, University Park, Pennsylvania, United States of America; 3 Northwestern University Feinberg School of Medicine Department of Pathology, Northwestern University, Chicago, Illinois, United States of America; Virgen Macarena University Hospital, School of Medicine, University of Seville, SPAIN

## Abstract

**Background:**

Gestational diabetes mellitus (GDM) is associated with increased placental weight and the presence of placental malperfusion lesions, likely related to high blood glucose. The relationship between high glucose without overt GDM, and placental characteristics is not well understood.

**Objective:**

To examine the relationships between glucose challenge test (GCT) concentrations, GDM, and placental characteristics associated with GDM.

**Methods:**

We conducted a secondary analysis of medical record data from singleton placentas sent to pathology at Northwestern Memorial Hospital (2011–2022; n = 11,585). Placentas were submitted based on standard clinical protocol. Data included maternal demographic variables, GCT concentrations, GDM diagnosis, placental weight, and vascular malperfusion lesions (accelerated villous maturation, increased syncytial knots, delayed villous maturation, and increased perivillous fibrin deposition). We classified GCT <140 mg/dL as pass and ≥140 mg/dL as fail. GDM was classified by diagnosis. We categorized glucose groups into *pass GCT/no GDM*, *fail GCT/no GDM*, and *GDM*. We used linear and Poisson (due to non-convergence of log-binomial) regression models to examine the association between GCT concentrations or groups with placental outcomes, adjusting for maternal age, race and ethnicity, parity, gestational age at delivery, and infant sex.

**Results:**

Of placentas sent to pathology, 17% were in the *fail GCT/no GDM* group and 5% were in the *GDM* group. Compared to the *pass GCT/no GDM* group, the adjusted mean placental weight was heavier by 13.6 grams [95% CI: 8.8, 18.3] in the *fail GCT/no GDM* and 22.0 grams [13.8, 30.2] in the *GDM* group. Patients diagnosed with GDM had a 36% [2%, 81%] increased adjusted risk of delayed villous maturation compared to the *pass GCT/no GDM*. The risk of the other lesions (accelerated villous maturation, increased syncytial knots, and increased perivillous fibrin deposition) was not significantly different between groups.

**Conclusion:**

GDM and high glucose concentrations without GDM were associated with heavier placentas. Patients with GDM had a higher risk of delayed villous maturation, but risk of other placental lesions was similar.

## Introduction

Gestational diabetes mellitus (GDM) is defined as hyperglycemia or glucose intolerance with first occurrence during pregnancy, and, of importance, they must have no history of type 1 or type 2 diabetes mellitus [1]. A systematic review and meta-analysis examined 1,550,917 subjects in the United States and Canada, of which the mean prevalence of GDM was 6.9% [2]. In 2021, 16.9 million pregnancies were classified with GDM globally [2]. GDM can result in several adverse pregnancy outcomes, including miscarriage, prematurity, and Cesarean section [3–6]. Of note, GDM typically resolves at delivery, yet five years after pregnancy, the risk of type 2 diabetes mellitus increases by seven-fold in the mother [7]. Additionally, exposure to hyperglycemia in utero is associated with fetal hyperinsulinemia [8], which could result in insulin resistance in childhood [9]. Consequently, GDM can have acute and long-term health implications for the pregnant person and child.

The placenta supports the growth and development of the fetus [10]. Past studies consistently report higher placental weight in GDM pregnancies, when compared to non-GDM pregnancies [11–45]. Additionally, histological analysis has found an increase in accelerated villous maturation, increased syncytial knots, delayed villous maturation, and increased perivillous fibrin deposition in GDM, type 1 diabetes, and/or type 2 diabetes mellitus pregnancies [25,46–50]. GDM is linked with increased maternal and fetal vascular malperfusion, which includes the above lesions and villous infarcts, fibrinoid necrosis, villous edema, and other complications [51–54].

An individual with poorly controlled hyperglycemia may have impairment of villi development, thus resulting in malperfusion lesions [25,46–49]. Notably, the underlying physiology that results in GDM-induced placental changes remains to be uncovered. Past literature has established a relationship between GDM and some placental changes, namely, weight. Still, these comparisons are often limited to binary groups of non-GDM controls and overt GDM cases [11–45]. The relationship between placental changes and glucose concentrations on a continuum has not been examined to our knowledge.

One method of diagnosing GDM is a two-step strategy: a non-fasted 50-gram glucose challenge test (GCT; screening measure), which, if failed, proceeds to an oral glucose tolerance test (OGTT; diagnostic measure) [55]. Notably, a pregnant person may fail the GDM screening but pass the GDM diagnostic, often referred to as pre-GDM or one abnormal value. Two studies have examined placental outcomes in pregnancies with pre-GDM [56,57]. *Nataly et al.* reported higher proportions of fetal vascular malperfusion lesions in patients with pre-GDM compared to patients with overt GDM [57]. *Rudge et al.* reported a higher proportion of syncytial knots in patients with pre-GDM than in patients with normoglycemia [56]. Additionally, patients with pre-GDM had higher proportions of intervillous fibrosis and delayed villous maturation compared to patients with normoglycemia or overt GDM, albeit not statistically significant. Both studies provide valuable insights into the relationship between glucose and the placenta; however, gaps remain in our understanding. The present study addresses methodological gaps of past work by examining a large obstetric sample with a non-GDM control group.

Given the limited data examining glucose as a continuous variable, in particular high glucose without GDM, we aimed to examine the relationships between GCT concentrations, GDM, and placental characteristics, including placental weight and placental vascular malperfusion lesions (accelerated villous maturation, increased syncytial knots, delayed villous maturation, and increased perivillous fibrin deposition). We hypothesized that pregnant patients with high GCT concentrations and those who fail their GCT but “pass” full GDM diagnostic tests will have similar placental characteristics (higher placental weight, more malperfusion lesions) to pregnant patients diagnosed with overt GDM.

## Methods

We conducted a retrospective cohort study of anonymized medical record data from Northwestern Memorial Hospital (affiliated with Northwestern University; Chicago, Illinois) between January 1, 2011 to December 31, 2022. Northwestern Memorial Hospital is a tertiary-level hospital that performs approximately 10,000 deliveries per year, of which about 20% of delivered placentas are sent to pathology. Medical chart extraction was conducted as part of an existing multi-institution study with ethical approval through a single-IRB protocol at The Pennsylvania State University (IRB: STUDY00020697). Northwestern University was a participating site with a reliance agreement under the single-IRB.

At the time of medical service, patients sign a consent form for treatment, which includes an acknowledgement that their data may be utilized for research purposes. For the present study, patients were not required to sign an additional consent form, as we received a waiver of consent from the IRB. One study investigator at Northwestern (JAG) conducted all data extraction and de-identification from October 17, 2023, until April 18, 2025; no other authors had access to or could identify participants during or after data collection.

### Patient population

All pregnancies within the medical record extraction period were eligible for inclusion if they had a complete pathology report. Placentas sent to pathology are required to meet a certain set of criteria, according to Northwestern Memorial Hospital guidelines. The Northwestern Memorial Hospital decision tree for placental pathology assessment is available in the *Supplementary Material*s (S1 Fig). Briefly, placentas can be sent to pathology if they meet criteria for placental (e.g., infarct, cord knot, etc.), fetal (premature < 34 weeks, stillborn, etc.), maternal (severe PE, preterm premature rupture of membranes < 34 weeks, severe preeclampsia, etc.), and/or newborn (Apgar score ≤ 6 at five minutes, ventilatory assistance > 10 minutes, etc.) abnormalities. Of note, GDM is not a criterion for a placental pathology assessment.

Along with a complete pathology report, patients were required to meet the following criteria: singleton pregnancy, completed the GCT (GDM screening) and gestational age at birth ≥ 140 days (20 weeks, to exclude miscarriage). The exclusion criterion was a history of diagnosed type 1 or type 2 diabetes mellitus.

**Fig 1:**
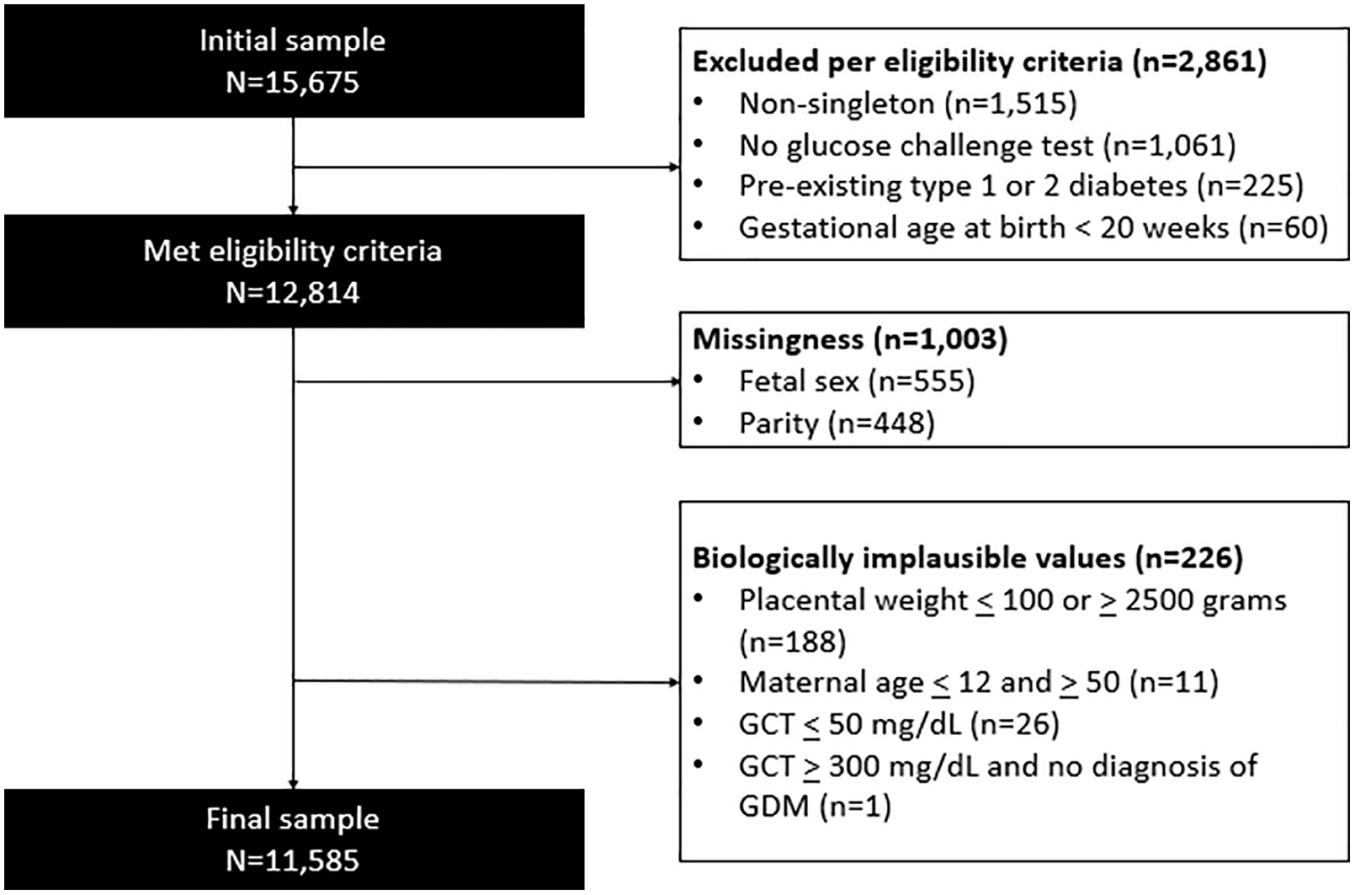
Study selection of pregnant patients from Northwestern Memorial Hospital. Abbreviations: GDM = gestational diabetes mellitus.

Our initial sample included 15,675 pregnancies (**Fig 1**). A total of 2,861 were removed per our eligibility criteria, 1,003 were removed due to missingness, and 226 were removed due to biologically implausible values (i.e., placental weight, maternal age, GCT). The final analytic sample was 11,585. We conducted power analysis to determine the power for multivariate analysis given our sample size (n = 11,585), for linear, logistic, and Poisson regression [58]. After accounting for at least ten covariates and one degree of freedom, we were powered at 100% for multivariate linear, log-binomial, and Poisson regression, at a significance level of alpha = 0.05 and a large effect size. Models and details on the statistical plan are in *Statistical Analysis.*

### Covariates

Demographic variables of interest included maternal age, race and ethnicity (see S1 Supporting Information), parity, gestational age at delivery, infant sex, and maternal hypertension. Maternal age was reported in whole years, parity was patient-reported as the number of births before the current pregnancy, gestational age at delivery was recorded per hospital procedures (last menstrual period and/or first-trimester ultrasound scan), and infant sex was recorded as male, female, or unknown (we recoded as missing as there was no distinction as intersex or other). Infant birth weight was extracted from the medical record. We did not have access to maternal height, pre-pregnancy or pregnancy weights, or body mass index (BMI; kg/m^2^) data. We classified maternal hypertension based on the International Classification of Diseases, 10^th^ Revision, Clinical Modification (ICD-10-CM) diagnosis codes in the patient’s medical record (**S1 Table**) [59].

### Glucose and GDM

We identified patients with GDM based ICD-10-CM diagnosis codes in the medical record, detailed in our Supplementary Materials (**S2 Table**) [59]. We classified GDM: (1) GDM, diet control, (2) GDM, insulin control, (3) GDM, oral drug control, and (4) GDM, unspecified control. We grouped all categories to determine the total diagnosis of GDM for the entire study population. Diagnostic terms were not mutually exclusive and total diagnosis of GDM was determined if any of the above categories were present in an individual patient.

Our first exposure of interest was the glucose concentrations from GCT for every patient, with and without GDM, on a continuum*.* The GCT is a non-fasted screening test, or step one of a two-step strategy, to determine if a patient requires a formal OGTT (diagnostic of GDM) [55]. It consists of a 50-gram glucose drink followed by a blood sample taken one hour post consumption. Our second exposure of interest was three “glucose groups” created based on a patient’s (1) GCT results and (2) diagnosis of GDM (**Table 1**). These groups were created to capture a middle group between non-GDM and GDM that had high glucose in the GCT screening but were not diagnosed with GDM. Of note, 88 participants had a “passing” GCT value but were diagnosed with GDM, likely related to clinical decisions not captured in the medical record data extracted. Those individuals were grouped with participants who were diagnosed with GDM to comprise the *GDM* group.

**Table 1:** Glucose groups based on GCT concentration and GDM diagnosis.

Groups	GCT result (mg/dL)	Diagnosed with GDM?
Pass GCT/No GDM	< 140	No
Fail GCT/No GDM	≥ 140	No
GDM	≥ 140^†^	Yes

† n = 88 had a GCT result < 140 mg/dL but had a GDM diagnosis and were therefore classified in the GDM group. Abbreviations: GCT = glucose challenge test; GDM = gestational diabetes mellitus

Two patients had a questionably high GCT (> 300 mg/dL). The first patient had a high GCT result (398 mg/dL) and did not have a GDM diagnosis, which suggested an error and was deemed biologically implausible. All models were run with and without this one erroneous value with nearly similar results. The investigative team discussed and concluded that this one record (GCT = 398 mg/dL) would be excluded from the primary analysis. The second patient had a high GCT (376 mg/dL) and a GDM diagnosis, which we considered plausible. This patient was kept in the primary analysis, and we conducted a sensitivity analysis excluding the second patient.

### Placental pathology report

We extracted placenta data from the patient’s placental pathology report; perinatal pathologists write all reports following a standardized protocol per Northwestern Memorial Hospital guidelines. Outcomes were placental weight (weighed in the gross exam after trimming back membranes and cutting the umbilical cord < 1 cm from the disc), accelerated villous maturation, increased syncytial knots, delayed villous maturation, and increased perivillous fibrin deposition. Accelerated villous maturation, increased syncytial knots, and delayed villous maturation are defined per the Amsterdam Placental Workshop Group [60]. Perivillous fibrin deposition (also known as massive perivillous fibrin deposition) is characterized by *Faye-Petersen and Ernst* [61]. Medical records may have varied terms for perivillous fibrin deposition. Per the guidance of our placental pathologist (JAG), we combined four lesion diagnoses to characterize increased perivillous fibrin deposition: increased perivillous fibrin, syndromes of perivillous fibrin deposition, massive perivillous fibrin deposition, and borderline massive perivillous fibrin deposition. Our investigative team selected these lesions as they are commonly found in placentas with diabetes [25,46–50].

### Statistical analysis

We used kernel density plots with a normal curve overlay to visualize the normality of distributions for continuous variables. Median, skewness, and kurtosis were examined as well. Placental weight was normally distributed and GCT was close to normal and examined without log transformation [62]. We examined frequencies of discrete variables. We looked for a non-linear relationship between the continuous exposure (GCT) and continuous outcome (placental weight) using LOWESS (Locally Weighted Scatterplot Smoot) plots and found that it was linear.

We assessed demographic characteristics by glucose groups with one-way ANOVA and pairwise t-test for continuous variables with normal distributions (maternal age) and Kruskal-Wallis rank sum test and Dunn’s pairwise test for continuous variables with non-normal distributions (gestational age at birth (days) and infant birth weight (grams)), with values reported as medians with interquartile ranges (IQR) [63–65]. We used Pearson’s chi-squared test and proportional pairwise test for categorical variables (race and ethnic group, parity, infant sex, preterm birth) [66,67]. We conducted all pairwise tests with Bonferroni correction [68].

#### Main analysis.

We used linear regression models to estimate the unadjusted and adjusted association of glucose groups and GCT concentrations (each exposure in a separate model) with placental weight. We then estimated the unadjusted relative risk (RR) and adjusted relative risk (ARR) for the association of GCT values and glucose groups with the categorical placental outcomes (accelerated villous maturation, increased syncytial knots, delayed villous maturation, and increased perivillous fibrin deposition). We initially tested log-binomial regression models; however, models would not converge and Poisson regression with robust standard errors was used instead [69,70]. All models were adjusted for maternal age, maternal race and ethnicity, parity, gestational age at birth, and infant sex. For all adjusted analyses, we collapsed race and ethnicity into three groups (NH White, NH Black or African American, and all other groups) and parity into two groups (0/ ≥ 1) to ensure model stability and to improve model interpretability. We adjusted for all relevant covariates simultaneously rather than determining inclusion based on individual variable contributions to the model.

#### Interactions and sensitivity analysis.

We examined interactions in these associations by infant sex (male/female) and parity (0/ ≥ 1). We considered an interaction with a *p* value < 0.1 to be statistically significant, similar to prior literature, [71–73] and examined differences to determine if they were meaningful. For interactions with glucose groups (2-category variable x 3-category variable), we used a Wald test to calculate the *p* value of the combined interaction terms post-estimation [74–76].

In sensitivity analysis, we examined all unadjusted and adjusted models without patients diagnosed with hypertension (defined in **S1 Table**), to test the influence of hypertension on our results.

All statistical tests were two-sided, and we considered results statistically significant if a *p* value was < 0.05. All statistical analyses were run in R, version 4.4.1 [77].

## Results

**Table 2** presents the population characteristics according to different glucose groups. Mean participant age and gestational age at delivery were similar across groups, but statistically different. Participants predominantly self-identified as NH White (*pass GCT/no GDM* = 57%; *fail GCT/no GDM* = 52%; *GDM* = 39%) and were delivering for the first time (*pass GCT/no GDM* = 65%; *fail GCT/no GDM* = 64%; *GDM* = 51%). Median glucose concentrations from the GCT were higher than *pass GCT/no GDM* group, by approximately 50 mg/dL. Just over half of the patients delivered male infants. Infant birthweight was highest in the *pass GCT/no GDM* group, but not statistically different across groups. Mean gestational age was highest in the *pass GCT/no GDM* group. Preterm birth (< 259 days) was proportionally highest in the *fail GCT/no GDM* group.

**Table 2:** Maternal and infant characteristics by glucose groups (n = 11,585).

Category	Pass GCT/no GDM (n = 9,018)	Fail GCT/no GDM (n = 1,984)	GDM (n = 583)	*p* value*
**Maternal**				
**Maternal age (years)**	33 (5)^a^	34 (5)^b,c^	34 (5)^b,c^	< 0.001
**Race and ethnic group, n (%)**				< 0.001
NH White	5,124 (57%)^a^	1,022 (52%)^b^	225 (39%)^c^	
NH Black or African American	1,136 (13%)^a,c^	184 (9%)b,c	67 (11%)^c^	
NH Asian	704 (8%)^a^	256 (13%)^a^	104 (18%)^b^	
Hispanic or Latino and > 1 race^†^	485 (5%)^a^	118 (6%)^a^	48 (8%)^a^	
Other^‡^	44 (0.5%)^a^	8 (0.4%)^a^	4 (0.7%)^a^	
Unknown	1,525 (17%)^a^	396 (20%)^b^	135 (23%)^b^	
**Parity, n (%)**				< 0.001
0	5,840 (65%)^a^	1,271 (64%)^a^	297 (51%)^b^	
1	2,188 (24%)^a^	469 (24%)^a^	183 (31%)^b^	
2 or greater	990 (11%)^a^	244 (12%)^a^	103 (18%)^b^	
**Glucose challenge test, mg/dL, median (IQR)**	105 (29)	153 (20)	159 (34)	N/A^**#**^
**Infant**				
**Gestational age at birth (days), median (IQR)**	273 (19)^a^	271 (20)^c^	269 (16)^c^	< 0.001
**Male, n (%)**	4,649 (52%)	1,066 (54%)	305 (52%)	0.203
**Birth weight, grams, mean (SD)** ^§^	3,036 (674)	3,023 (748)	3,029 (681)	0.999
**Preterm birth, n (%)**	1,886 (21%)^a^	514 (26%)^b^	144 (25%)^a,b^	< 0.00_1_

All percentages represent the total proportion in each glucose group, not the total sample

Different letters indicate significant differences (*p* < 0.05) in pairwise tests

* One-way ANOVA and pairwise t-test for continuous variables with normal distributions (maternal age); Kruskal-Wallis rank sum test and Dunn’s pairwise test for continuous variables with non-normal distributions (gestational age at birth (days)); Pearson’s chi-squared test and proportional pairwise test for categorical variables (race and ethnic group, parity, infant sex, preterm birth). All pairwise tests were conducted with Bonferroni correction

† Defined as American Indian and Alaska Native, Black or African American, Asian, Native Hawaiian or Pacific Islander, and White; represent < 1% of the total population

‡ Defined as American Indian and Alaska Native, Native Hawaiian or Pacific Islander (NH or unknown ethnicity); represent < 1% of the total population. Pairwise comparison must be interpreted with caution due to the small sample size between groups (n < 30)

# Glucose challenge tests defined glucose groups; thus, not part of group comparisons

§ Missing data: pass GCT/no GDM = 99; fail GCT/no GDM = 20; GDM = 6

Abbreviations: GCT = glucose challenge test; GDM = gestational diabetes mellitus; NH = Non-Hispanic; SD = standard deviation

### Glucose concentrations and placental outcomes

**Fig 2:**
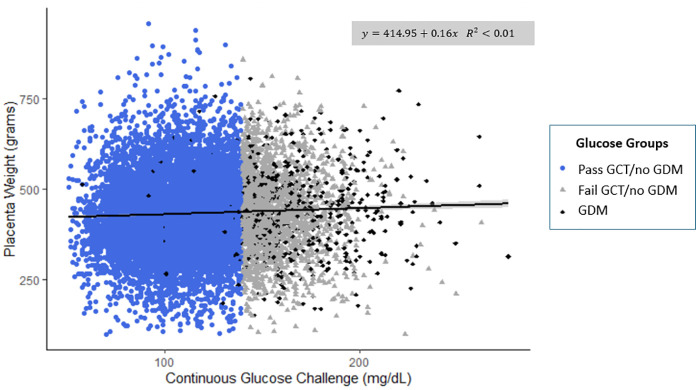
Continuous glucose challenge concentrations (mg/dL) and placental weight (grams) stratified by glucose groups (n = 11,583). Excluded from figure: placental weight > 1200 grams (n = 1); glucose concentrations > 300 mg/dL (n = 1). Abbreviations: GCT = glucose challenge test; GDM = gestational diabetes mellitus.

The range of placental weights was 100–1,265 grams. GCT concentrations had a slight positive relationship with placental weight (**Fig 2**). A 10 mg/dL increase in GCT was associated with a 1.63 gram increase in placental weight (95% CI: 0.95, 2.30, *p* value < 0.001). After adjustment, a 10 mg/dL increase in GCT was more strongly associated with placental weight (3.26 gram; 95% CI: 2.66, 3.86), *p* value < 0.001). The relationship between GCT and placental lesions was non-significant across all models (**S3 Table**).

### Glucose groups and placental characteristics

A total of 78% of patients were in the *pass GCT/no GDM* group, 17% were in the *fail GCT/no GDM* group, and the remaining 5% were in the *GDM* group*.* **S4 Table** shows the frequency of each GDM diagnostic group, with the most prominent diagnosis being *diet control* (n = 427, followed by *unspecified control* (n = 401). Importantly, diagnoses are not mutually exclusive. Mean (SD) placental weight was highest in the *GDM* group (**Table 3**; 446 grams (111)). Pregnant patients diagnosed with *GDM* had a significantly higher mean difference in placental weight than those in the *pass GCT/no GDM* group (unadjusted mean difference: 14.1 grams (95% CI: 4.9, 23.4; **Table 3**). This difference was higher after adjustment (AMD: 22.0 grams (95% CI: 13.8, 30.2). Pregnant patients in the *fail GCT/no GDM* group had a mean difference in placental weight that was nearly 14 grams higher when compared to the *pass GCT/no GDM* group after adjustment (AMD: 13.6 grams (95% CI: 8.8, 18.3)).

**Table 3:** Associations between glucose groups and placental weight (in grams) with interactions by infant sex (n = 11,585).

		Unadjusted		Adjusted†	
Variable	Mean (SD)	Mean difference (95% CI)	*p* value	Mean difference (95% CI)	*p* value
**Pass GCT/no GDM**	432 (110)	Reference			
**Fail GCT/no GDM**	438 (114)	5.9 (0.6, 11.3)	0.030	13.6 (8.8, 18.3)	< 0.001
**GDM**	446 (111)	14.1 (4.9, 23.4)	0.003	22.0 (13.8, 30.2)	< 0.001
	**Female** ^‡^				
**Pass GCT/no GDM**	430 (108)	Reference			
**Fail GCT/no GDM**	442 (114)	12.2 (4.3, 20.0)	0.002	19.0 (12.1, 26.0)	< 0.001
**GDM**	445 (111)	14.8 (1.4, 28.2)	0.030	24.9 (13.1, 36.7)	< 0.001
	**Male** ^‡^				
**Pass GCT/no GDM**	434 (111)	Reference			
**Fail GCT/no GDM**	434 (113)	0.4 (−6.9, 7.8)	0.908	8.7 (2.3, 15.2)	0.008
**GDM**	447 (111)	13.5 (0.7, 26.3)	0.039	19.3 (8.0, 30.6)	0.001

† Adjusted for maternal age, maternal race/ethnicity, parity, gestational age at birth, and infant sex

‡ Unadjusted models for interactions include infant sex and interactions by infant sex

Race and ethnicity variable was categorized into three groups: NH White, NH Black or African American, and all other race and ethnic groups

Parity variable was categorized into two groups: 0 and ≥ 1

Wald test *p value* = 0.094 for interactions between glucose groups and infant sex in adjusted model

Abbreviations: CI = confidence intervals; GDM = gestational diabetes mellitus; GCT = glucose challenge test; NH = non-Hispanic; SD = standard deviation.

**Fig 3:**
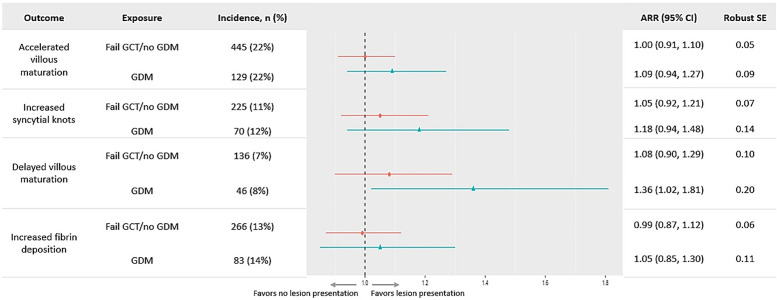
Adjusted relative risk of vascular malperfusion lesions by glucose groups (n = 11,585). Circle = Fail GCT/no GDM; Triangles = GDM; Reference glucose group: pass GCT/no GDM; All values were adjusted for maternal age, race, parity, gestational age at birth, and infant sex. Abbreviations: ARR = adjusted relative risk; CI = confidence interval; GCT = glucose challenge test; GDM = gestational diabetes mellitus; NH = non-Hispanic; RR = relative risk; SE = standard error.

In unadjusted models, accelerated villous maturation risk was 13% (95% CI: 3%, 24%) higher in the fail *GCT/no GDM* group when compared to the *pass GCT/no GDM* group (**S5 Table**). This association was attenuated after adjustment (ARR: 1.00 (0.91, 1.10)). Delayed villous maturation risk was 36% higher in the *GDM* group when compared to the *pass GCT/no GDM* group after adjustment (95% CI: 2%, 81%; **Fig 3**). We found no difference in risk for all other associations of GCT groups and placental lesions.

### Interaction analysis

The relationship between glucose groups and placental weight differed by infant sex (**Table 3**; *p* value = 0.094). Both females and males had heavier placentas in the fail GCT/no GDM and GDM groups, compared to pass GCT/no GDM. For females, the difference between weights in fail GCT/no GDM and pass GCT/no GDM was greater than in males (19.0 vs 8.7), which may indicate different placental adaptations [78–81]. We did not observe an interaction by parity (Wald test *p* value = 0.164).

Sensitivity analysis found no difference in adjusted models for both continuous and categorical exposures when the one elevated GCT (> 300 mg/dL) was excluded. Results were similar for nearly all models when patients with hypertension were excluded (**S6****-****S9 Tables**). The association between GDM and delayed villous maturation diminished (**S9 Table**), but the spread was similar to the primary analysis.

## Discussion

In this retrospective cohort study of anonymized medical record data from Northwestern Memorial Hospital, we found that GCT on a continuum had a positive relationship with placental weight; but was not associated with risk of placental malperfusion lesions. A failed GCT was associated with higher placental weight in both female and male infants, with and without GDM. Notably, the relationship was strengthened between glucose groups and placental weight after adjustment for covariates. Patients with GDM had a greater risk of delayed villous maturation compared to patients who passed their GCT and were not diagnosed with GDM. Glucose groups were not associated with any other placental malperfusion lesions after adjustment.

Our results reported a very small positive relationship between continuous GCT and placental weight. Both glucose groups were associated with heavier placentas, suggesting that hyperglycemia during pregnancy is positively associated with placental weight, irrespective of a GDM diagnosis. Past evidence suggests that GDM is associated with heavier placentas and lower placental efficiency [11–45, 82]. Additionally, a larger placenta may be an important adaptation to support fetal weight [83]. Our finding of a heavier placenta in individuals with a high GCT but no GDM suggests placental adaptations even at moderately high glucose exposure. The clinical relevance of modest placental weight differences in relation to maternal and fetal outcomes should be investigated.

Unlike GCT, the relationship between GDM and increased placental weight has been well-established in past literature [11–45]. *Barke et al.* conducted a case-control study and reported a 44 grams higher unadjusted mean placental weight in GDM pregnancies, though not statistically significant, [12], it is much higher than the 22 g difference we observed. In a separate case-control study, *Magee et al.* reported a 120-gram higher mean unadjusted placental weight in GDM pregnancies when compared to non-GDM pregnancies [32]; again, higher than our results. Similar to our research, both studies were conducted in tertiary-level hospitals in the United States and included a non-GDM control group. *Barke et al.* examined placentas sent to pathology per physician discretion (similar to our study), and *Magee et al.* selected participants without pregnancy complications (unlike our study), which may have contributed to the larger difference observed. In contrast to our research, both studies were small (n < 50 for both studies) and did not account for GCT on a continuum [12,32]. Additionally, they did not include an examination of placental malperfusion lesions.

Unlike our findings, some recent literature reported lower mean placental weight in GDM pregnancies than in non-GDM pregnancies [84,85]. One study by *Hiden et al*.reported but did not test for differences between placental weights (GDM vs the control), and they had a small sample size (n < 30) [84], and *Kadivar et al.* found no statistical difference between mean placental weights by GDM status [85], although their difference (an 18-gram mean placental weight difference) is similar to that of the current study. Both studies examined GDM as binary classification, presented unadjusted mean placental weights, and did not examine the relationship between a failed GCT and placental weight. Lastly, *Hiden et al.* examined pregnant patients undergoing cesarean section, and *Kadivar et al.* sampled from a prospective obstetric cohort.

Interaction analysis by infant sex found higher placental weight, if a patient had a failed GCT, in both female and male infants, however, female infants consistently had higher mean placental weight differences compared to males. Unlike females, male placental weights only increased in the *GDM* group, when compared to the *pass GCT/no GDM*. These results suggest that female births may be more sensitive to the maternal environment, compared to male births. Past literature has reported differences in placental size by infant sex, during famine, and periods of fasting (i.e., Ramadan) with contradicting results [86,87]. Studies examining placental efficiency by sex in adverse environments (e.g., asthma, preeclampsia, etc.) have found that females are born smaller than males, but females may have greater placental reserve capacity [78,88,89]. The mechanisms driving sex-specific differences are not well understood. Past evidence suggests greater placental efficiency for male infants, but female infants have placentas better able to adapt to the *in utero* environment [78–81]. Such findings may be related to differences in long-term health outcomes in male versus female offspring; however, the relationship with placental weight is unclear. *Christians and Chow* conducted a large multi-site cohort study and reported a five-gram greater mean placental weight in males, compared to females, although not significant [90]. Importantly, the investigators did not examine placental differences by maternal glucose or GDM. Cumulatively, infant sex differences in placental adaptations exist in adverse pregnancy environments, but this relationship to placental weight requires further investigation.

The mechanisms driving glucose concentrations and placental weight are not well understood and are largely reported in pregnancies with type 1 diabetes and/or type 2 diabetes [91–93]. *Nelson et al.* reported an increased volume of the intervillous space in pregnancies with type 1 diabetes when compared to controls [91]. *Jauniaux and Burton* also reported higher intervillous space volume, along with an increase in trophoblast and placental volume in patients with type 1 diabetes, when compared to healthy controls [92]. Lastly, *Higgins et al.* reported greater terminal villi surface area and immature intermediate villi in pregnancies with type 1 or type 2 diabetes, compared to healthy controls without diabetes [93]. Additionally, capillary surface area was increased in patients with type 1 diabetes compared to the control. Cumulatively, higher maternal glucose may increase intervillous space volume, placental volume, villi surface area, and capillary surface area. Importantly, all studies were conducted in patients with type 1 and/or type 2 diabetes. These populations should not be generalized to GDM pregnancies, as diabetes was present during periconception and the earliest events of placentation. Instead, these findings may provide context for studies examining placental development in GDM pregnancies.

### Accelerated Villous Maturation

Our results did not find a relationship between high GCT and accelerated villous maturation in adjusted models. Accelerated villous maturation is within the overarching pathology of maternal vascular malperfusion and is characterized by inadequate spiral artery remodeling and thus poor blood flow [54,94]. Past literature does not have a clear consensus on the relationship between accelerated villous maturation and hyperglycemia, partly due to heterogeneity in study designs. *Siassakos et al.* examined placentas sent to pathology and reported an increased incidence of accelerated villous maturation in patients with any abnormal glucose reading in their OGTT [95]. Interestingly, patients with GDM had a lower incidence of accelerated villous maturation. Investigators did not describe the role of dietary and medication intervention in GDM patients, and their samples were small. A recent study from *Goto et al.* examining placentas sent to pathology, reported no presence of accelerated villous maturation in GDM placentas, compared to the control [25]. The proportion of maternal vascular malperfusion was greater in the control group, compared to GDM. Importantly, *Goto et al.* examined patients who failed their GCT but passed their OGTT. Additionally, neither study examined GCT on a continuum.

### Syncytial Knots

Our results also did not find a relationship between high GCT and increased syncytial knots in unadjusted and adjusted models. Syncytial knots are characteristic of accelerated villous maturation and are identified by increased syncytial nuclei at the terminal villi [94,96]. Syncytial knots are an expected pathology with an average proportion of nearly 30% of villi in a term placenta; however, increased syncytial knots can be a sign of immaturity or malperfusion [96]. Increased syncytial knots for gestational age are likely a compensatory mechanism in placental formation to maximize the transfer of nutrients to the fetus [96]. The association between GDM and increased syncytial knots has been well-established in past literature [49,97–100]. *Aldahmash, Alwasel, and Aljerian* reported a greater proportion of increased syncytial knots in pregnancies with GDM compared to the control [48]. Similarly, *Dasgupta et al.* reported a greater incidence of increased syncytial knots that were higher in pregnancies with GDM to the control [47]. Both studies were case-control, had similar sample sizes, and defined increased syncytial knots as more than 30–33% of villi in the placenta [60,101]. Of note, *Dasgupta et al.* examined placentas that were sent to pathology – as such pregnancies with GDM were not compared to a healthy, normal obstetric population. In contrast, *Bhattacharjee et al.* conducted a cross-sectional analysis of placentas sent to pathology and reported no significant difference in increased syncytial knots between pregnancies with GDM compared to the control [102]. Patients with mild hyperglycemia (defined as normal OGTT with an altered glucose profile) had greater syncytial knots, albeit not significant, when compared to the control.

Our results reported an increased risk of delayed villous maturation in pregnancies with GDM when compared to the control. Delayed villous maturation is a supportive finding of fetal vascular malperfusion and is characterized by a reduction in the critical vascular branching of the chorionic villi and thus a reduction in vasculosyncytial membrane formation [52,53,103,104]. A failed GCT without overt GDM was not associated with an increased risk of delayed villous maturation in unadjusted or adjusted models. Past literature supports the relationship between delayed villous maturation and GDM [98,99,102,105,106]. Recent evidence suggests an association between abnormal glucose values, without GDM, and delayed villous maturation. *Nataly et al.* reported an increased incidence of fetal vascular malperfusion lesions in their OAV group, compared to patients with GDM receiving medication therapy. In contrast, our study found no relationship between GCT on a continuum or a failed GCT without GDM and select fetal malperfusion lesions (delayed villous maturation and increased perivillous fibrin deposition). Of note, *Nataly et al.* examined patients with an abnormal value in their OGTT, whereas our study examined a failed GCT (glucose screen before the OGTT). Findings may suggest further investigation into abnormal values in the OGTT itself and the risk of vascular malperfusion lesions. *Rudge et al.* examined patients with mild gestational hyperglycemia (MGH; defined as a normal OGTT with at least two borderline values) [56]. Placental dysmaturity (not defined) was present in patients with MGH, GDM, and overt diabetes, but not in the control group. Although non-significant, MGH can lead to the incidence of placental lesions. Importantly, sample comparisons were unbalanced, with six patients with normoglycemia, eight patients with GDM, 34 with MGH, and 83 with ‘overt’ diabetes (not defined).

Finally, our results found no associations between glucose groups and perivillous fibrin deposition. Perivillous fibrin deposition is characterized by excessive fibrin and trophoblasts surrounding the terminal villi [107]. An increase in fibrin deposition is a criterion for global partial fetal vascular malperfusion, which is linked to obstructions to the umbilical cord [52]. The relationship between glucose concentrations, specifically GDM, and increased perivillous fibrin deposition is inconsistent. This may be partly due to the different locations of fibrin deposition in the placenta (intramural versus intervillous versus perivillous) [25,47,48]. Importantly, these lesions should not be grouped and generalized when examining GDM pregnancies. Dasgupta et al. reported a significantly higher proportion of increased perivillous fibrin deposition in pregnancies with GDM compared to the control [47]. Similar to our methods, *Dasgupta et al.* conducted their study in a tertiary-level hospital and examined placentas sent to pathology. Unlike our study, the association between fibrin deposition and failed GCT was not examined.

Strengths of our study include that we extracted and analyzed a large medical record dataset from a high-resource, tertiary-level hospital. We examined placental outcomes from complete pathology reports with expert interpretation from an experienced placental pathologist (JAG). Additionally, we selected and examined lesions on the maternal *and* fetal sides of the placenta. Our study is not without limitations. All data were extracted from placentas sent to pathology; therefore, comparisons were not possible with a general obstetric population, introducing sampling bias. Our Fail GCT/no GDM group is an inherently heterogeneous sample of patients with expected variations in their OGTT results, potentially diluting our observed findings. The medical data we had access to did not include either the pre-pregnancy BMI or the ability to calculate it. BMI is an important confounder associated with GDM, and its absence from adjusted models is a key limitation in our analysis. We were unable to clearly determine whether GDM patients received dietary and/or pharmacological interventions during their pregnancy. If glucose was under good control with treatment, this may have limited the impact of GDM on the placenta compared to the control group. While our large sample size made the study overpowered to detect statistical differences, the statistically significant results were also, in our opinion, meaningfully different and findings on placental weight were consistent across analyses. Despite these limitations, our findings provide foundational knowledge on the relationship between glucose concentrations, placental morphology, and placental lesions.

## Conclusions

Our findings suggest that pregnant patients with elevated glucose concentrations have heavier placentas than those without elevated glucose during pregnancy. Interaction analysis found heavier placentas in females, compared to males, in patients with elevated glucose compared to patients without elevated glucose. These associations strengthened after adjustment. Patients with diagnosed GDM have an increased risk of delayed villous maturation, compared to the pass GCT/no GDM control. The relationships between glucose groups and placental malperfusion lesions (maternal and fetal) were otherwise null. Future studies are necessary to examine other placental morphological characteristics, such as placental diameter, volume, and central thickness. Future studies should also examine the clinical significance of patients who failed their GCT without overt GDM and their chronic disease risk postpartum (e.g., type 2 diabetes mellitus). The placenta is a complex organ that provides a window into pregnancy health, and the present study found that placentas are responsive to high glucose even in patients without a GDM diagnosis. Future research could investigate the potential for placental characteristics (such as weight) to aid in predicting and understanding health outcomes in both the pregnant person and the infant, particularly in settings where glucose testing is not possible or missed.

## Supporting information

S1 File Supporting Information. Methodology for the race and ethnicity variable. (DOCX)

S1 Fig Placental pathology decision tree from Northwestern Memorial Hospital. Abbreviations: APGAR = appearance, pulse, grimace, activity, and respiration; CMV = cytomegalovirus; DR = delivery room; HSV = herpes simplex virus; IUFD = intrauterine fetal demise; N = no; NICU = neonatal intensive care unit; PPROM = preterm premature rupture of membranes; SGA = small for gestational age; Y = yes.
(DOCX)

S1 Table Maternal hypertension categories by ICD-10-CM. All diagnoses were collapsed into the overall category “Maternal Hypertension”. Abbreviations: ICD-10-CM = International Classification of Diseases, 10th Revision, Clinical Modification; PE = preeclampsia; HTN = hypertension; HELLP: hemolysis, elevated liver enzymes, and low platelets; w/o=without.
(DOCX)

S2 Table Gestational diabetes mellitus diagnosis by ICD-10-CM. All diagnoses were collapsed into the overall category “GDM”. Abbreviations: ICD-10-CM = International Classification of Diseases, 10^th^ Revision, Clinical Modification; GDM = Gestational diabetes mellitus; Gestatnl diab in chldbrth ctrl by oral hypoglycemic drugs = Gestational diabetes in childbirth controlled by oral hypoglycemic drugs
(DOCX)

S3 Table Associations between glucose challenge tests (per 10 mg/dL increase) and placental lesions (n = 11,585). † Poisson regression model adjusted for maternal age, race and ethnicity, parity, gestational age at delivery, and infant sex. Abbreviations: ARR = adjusted relative risk; CI = confidence interval; RR = relative risk; SE = standard error.
(DOCX)

S4 Table GDM diagnostic criteria and total frequency. † Diagnoses are not mutually exclusive (i.e., some women have >1 GDM diagnosis). ‡ Only from the current pregnancy, history of GDM diagnoses not included. Abbreviations: GDM = gestational diabetes mellitus.
(DOCX)

S5 Table Associations between glucose groups and placental lesions (n = 11,585). The units for glucose challenge tests were 10 mg/dL. Interactions by infant sex and parity were not significant for any models (Wald test ≥ 0.1) and thus not included in this table. † Poisson regression model adjusted for maternal age, race (reference = NH White), parity (reference = 0), gestational age at delivery, and infant sex (reference = Female). Abbreviations: ARR = adjusted relative risk; CI = confidence interval; GCT = glucose challenge test; GDM = gestational diabetes mellitus; RR = relative risk; SE = standard error.
(DOCX)

S6 Table Associations between glucose challenge tests (per 10 mg/dL increase) and placental weight, a sensitivity analysis excluding patients diagnosed with maternal hypertension (n = 10,832). A total of 753 patients were diagnosed with maternal hypertension and were excluded. † Linear regression models were adjusted for maternal age, race and ethnicity, parity, gestational age at delivery, and fetal sex. Abbreviations: AMD = adjusted mean difference; CI = confidence interval; MD = mean difference.
(DOCX)

S7 Table Associations between glucose challenge tests (per 10 mg/dL increase) and categorical placental lesions (n = 10,832). A total of 753 patients were diagnosed with maternal hypertension and were excluded. † Poisson regression models were adjusted for maternal age, race and ethnicity, parity, gestational age at delivery, and fetal sex. Abbreviations: ARR = adjusted relative risk; CI = confidence interval; LGA = large for gestational age; RR = relative risk; SGA = small for gestational age.
(DOCX)

S8 Table Associations between glucose groups and placental weight, a sensitivity analysis excluding patients diagnosed with maternal hypertension (n = 10,832). A total of 753 patients were diagnosed with maternal hypertension and were excluded. † Linear regression model was adjusted for maternal age, race and ethnicity, parity, gestational age at delivery, and fetal sex. Abbreviations: CI = confidence intervals; GDM = gestational diabetes mellitus; GCT = glucose challenge test; NH = non-Hispanic; SD = standard deviation
(DOCX)

S9 Table Associations between glucose groups and categorical outcomes, a sensitivity analysis excluding patients diagnosed with maternal hypertension (n = 10,832). A total of 753 patients were diagnosed with maternal hypertension and were excluded. Poisson regression model was adjusted for maternal age, race and ethnicity, parity, gestational age at delivery, and fetal sex Abbreviations: CI = confidence intervals; GDM = gestational diabetes mellitus; GCT = glucose challenge test
(DOCX)
