# Supplementary material for: The relationship between maternal glucose concentrations, gestational diabetes mellitus, placental weight, and placental vascular malperfusion lesions: A retrospective study of a U.S. pregnancy cohort
Source: PLoS One. 2026 Mar 3;21(3):e0325415. doi: 10.1371/journal.pone.0325415 (PMC12956115; doi:10.1371/journal.pone.0325415)
Supplement: S1 File — (DOCX) [file pone.0325415.s001.docx]

**S1 Supporting Information. Methodology for the race and ethnicity variable**

*Coding and organization for race and ethnicity*

The medical record categorized race into two variables: their first identified race (Race 1), and their second identified race (Race 2). These variables were recategorized into the following: Asian (Asian, Asian Indian, Chinese, Filipino, Japanese, Korean, and Other Asian), Black or African American, White, Other (American Indian or Alaska Native, Native Hawaiian, Native Hawaiian or other Pacific Islander, Other Pacific Islander), and Unknown (Blank, Declined, None of the above, Patient declined to respond, Unable to answer, Unknown). The ethnicity variable included the following options: Not Hispanic, Hispanic, Declined, or Patient Unable to Respond. Multiple subcategories were available for those who identify as Hispanic (e.g., “Yes, Puerto Rican”, “Yes, Cuban”, etc.). If a participant reported Declined or Patient Unable to Respond for ethnicity, we recategorized it to 'unknown ethnicity.'

For our analysis, we classified race and ethnicity based on the Race 1 and Ethnicity variables as follows: Non-Hispanic (NH) White (includes White with unknown ethnicity), NH Black or African American (includes Black or African American with unknown ethnicity), NH Asian (includes Asian with unknown ethnicity), Hispanic or Latino (any races), Other, and Unknown. Other includes American Indian and Alaska Native (NH or unknown ethnicity) and Native Hawaiian or Pacific Islander (NH or unknown ethnicity), both of which make up < 1% of the total analytic sample. Unknown was used if a patient did not report their race and/or ethnicity (e.g., declined, unable to answer, etc.). The Race 2 variable was not included because < 1% had a designation (n=73).
