# Supplementary material for: The relationship between maternal glucose concentrations, gestational diabetes mellitus, placental weight, and placental vascular malperfusion lesions: A retrospective study of a U.S. pregnancy cohort
Source: PLoS One. 2026 Mar 3;21(3):e0325415. doi: 10.1371/journal.pone.0325415 (PMC12956115; doi:10.1371/journal.pone.0325415)
Supplement: S1 Fig — Abbreviations: APGAR = appearance, pulse, grimace, activity, and respiration; CMV = cytomegalovirus; DR = delivery room; HSV = herpes simplex virus; IUFD = intrauterine fetal demise; N = no; NICU = neonatal intensive care unit; PPROM = preterm premature rupture of membranes; SGA = small for gestational age; Y = yes. (DOCX) [file pone.0325415.s002.docx]

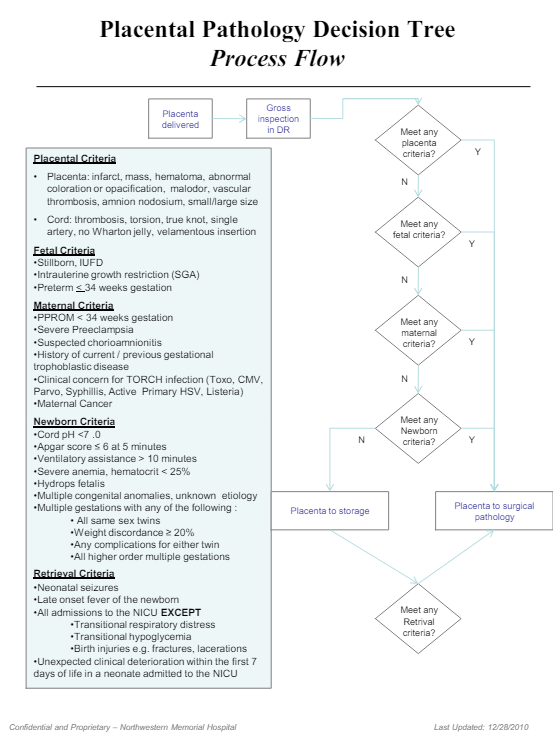


##### **S1 Fig. Placental pathology decision tree from Northwestern Memorial Hospital**

Abbreviations: APGAR= appearance, pulse, grimace, activity, and respiration; CMV=cytomegalovirus; DR=delivery room; HSV=herpes simplex virus; IUFD=intrauterine fetal demise; N=no; NICU=neonatal intensive care unit; PPROM=preterm premature rupture of membranes; SGA=small for gestational age; Y=yes
