# Supplementary material for: The relationship between maternal glucose concentrations, gestational diabetes mellitus, placental weight, and placental vascular malperfusion lesions: A retrospective study of a U.S. pregnancy cohort
Source: PLoS One. 2026 Mar 3;21(3):e0325415. doi: 10.1371/journal.pone.0325415 (PMC12956115; doi:10.1371/journal.pone.0325415)
Supplement: S1 Table — All diagnoses were collapsed into the overall category “Maternal Hypertension”. Abbreviations: ICD-10-CM = International Classification of Diseases, 10th Revision, Clinical Modification; PE = preeclampsia; HTN = hypertension; HELLP: hemolysis, elevated liver enzymes, and low platelets; w/o=without. (DOCX) [file pone.0325415.s003.docx]

| **S1 Table. Maternal hypertension categories by ICD-10-CM** | | |
| --- | --- | --- |
| **Diagnosis** | **ICD-10-CM Diagnosis** | **ICD-10-CM Code** |
| Eclampsia | Eclampsia, antepartum | O15.00 |
|  | Eclampsia complicating pregnancy, third trimester | O15.03 |
|  | Eclampsia, with delivery OR Eclampsia complicating labor | O15.1 |
|  | Eclampsia, with delivery, with current postpartum complication | O15.2 |
|  | Eclampsia complicating the puerperium | O15.2 |
|  | Eclampsia, unspecified as to time period | O15.9 |
| Pre-eclampsia | Mild to moderate pre-eclampsia, unspecified trimester | O14.00 |
|  | Mild to moderate pre-eclampsia, second trimester | O14.02 |
|  | Mild to moderate pre-eclampsia, third trimester | O14.03 |
|  | Mild to moderate pre-eclampsia, complicating childbirth | O14.04 |
|  | Mild to moderate pre-eclampsia, complicating the puerperium | O14.05 |
|  | Severe pre-eclampsia, unspecified trimester | O14.10 |
|  | Severe pre-eclampsia, second trimester | O14.12 |
|  | Severe pre-eclampsia, third trimester | O14.13 |
|  | Severe pre-eclampsia complicating childbirth OR Severe pre-eclampsia, with delivery | O14.14 |
|  | Severe pre-eclampsia, with delivery, with current postpartum complication | O14.15 |
|  | Unspecified maternal hypertension, first trimester | O16.1 |
|  | Unspecified maternal hypertension, second trimester | O16.2 |
|  | Unspecified maternal hypertension, third trimester | O16.3 |
|  | Unspecified maternal hypertension, complicating childbirth | O16.4 |
|  | Unspecified maternal hypertension, complicating the puerperium | O16.5 |
|  | Pre-existing hypertension with pre-eclampsia, second trimester | O11.2 |
|  | Pre-existing hypertension with pre-eclampsia, third trimester | O11.3 |
|  | Pre-existing hypertension with pre-eclampsia, complicating childbirth | O11.4 |
|  | Pre-existing hypertension with pre-eclampsia, complicating the puerperium | O11.5 |
|  | Pre-existing hypertension with pre-eclampsia, unspecified trimester | O11.9 |
| Pre-existing hypertension | Pre-existing essential hypertension complicating pregnancy, first trimester | O10.011 |
|  | Pre-existing essential hypertension complicating pregnancy, second trimester | O10.012 |
|  | Pre-existing essential hypertension complicating pregnancy, third trimester | O10.013 |
|  | Pre-existing essential hypertension complicating pregnancy, unspecified trimester | O10.019 |
|  | Pre-existing essential hypertension complicating childbirth | O10.02 |
|  | Pre-existing essential hypertension complicating the puerperium | O10.03 |
|  | Pre-existing hypertensive heart disease complicating pregnancy, first trimester | O10.111 |
|  | Pre-existing hypertensive heart disease complicating pregnancy, second trimester | O10.112 |
|  | Pre-existing hypertensive heart disease complicating pregnancy, third trimester | O10.113 |
|  | Pre-existing hypertensive heart disease complicating pregnancy, unspecified trimester | O10.119 |
|  | Pre-existing hypertensive heart disease complicating childbirth | O10.12 |
|  | Pre-existing hypertensive heart disease complicating the puerperium | O10.13 |
|  | Pre-exist hyp chronic kidney disease comp preg first tri OR Pre-existing hypertensive chronic kidney disease complicating pregnancy, first trimester | O10.211 |
|  | Pre-existing hypertensive chronic kidney disease complicating pregnancy, second trimester | O10.212 |
|  | Pre-existing hypertensive chronic kidney disease complicating pregnancy, third trimester | O10.213 |
|  | Pre-existing hypertensive chronic kidney disease complicating childbirth | O10.22 |
|  | Pre-existing hypertensive heart and chronic kidney disease complicating pregnancy, second trimester | O10.312 |
|  | Pre-existing hypertensive heart and chronic kidney disease complicating pregnancy, third trimester | O10.313 |
|  | Pre-existing secondary hypertension complicating pregnancy, first trimester | O10.411 |
|  | Pre-existing secondary hypertension complicating pregnancy, second trimester | O10.412 |
|  | Pre-existing secondary hypertension complicating pregnancy, third trimester | O10.413 |
|  | Pre-existing secondary hypertension complicating pregnancy, unspecified trimester | O10.419 |
|  | Pre-existing secondary hypertension complicating the puerperium | O10.43 |
|  | Unspecified pre-existing hypertension complicating pregnancy, first trimester | O10.911 |
|  | Unspecified pre-existing hypertension complicating pregnancy, second trimester | O10.912 |
|  | Unspecified pre-existing hypertension complicating pregnancy, third trimester | O10.913 |
|  | Unspecified pre-existing hypertension complicating pregnancy, unspecified trimester | O10.919 |
|  | Unspecified pre-existing hypertension complicating childbirth | O10.92 |
|  | Unspecified pre-existing hypertension complicating the puerperium | O10.93 |
|  | Pre-existing essential hypertension complicating pregnancy, first trimester | O10.011 |
|  | Pre-existing essential hypertension complicating pregnancy, second trimester | O10.012 |
|  | Pre-existing essential hypertension complicating pregnancy, third trimester | O10.013 |
|  | Pre-existing essential hypertension complicating pregnancy, unspecified trimester | O10.019 |
|  | Pre-existing essential hypertension complicating childbirth | O10.02 |
|  | Pre-existing essential hypertension complicating the puerperium | O10.03 |
|  | Pre-existing hypertensive heart disease complicating pregnancy, first trimester | O10.111 |
|  | Pre-existing hypertensive heart disease complicating pregnancy, second trimester | O10.112 |
|  | Pre-existing hypertensive heart disease complicating pregnancy, third trimester | O10.113 |
|  | Pre-existing hypertensive heart disease complicating pregnancy, unspecified trimester | O10.119 |
|  | Pre-existing hypertensive heart disease complicating childbirth | O10.12 |
|  | Pre-existing hypertensive heart disease complicating the puerperium | O10.13 |
|  | Pre-exist hyp chronic kidney disease comp preg first tri OR Pre-existing hypertensive chronic kidney disease complicating pregnancy, first trimester | O10.211 |
|  | Pre-existing hypertensive chronic kidney disease complicating pregnancy, second trimester | O10.212 |
|  | Pre-existing hypertensive chronic kidney disease complicating pregnancy, third trimester | O10.213 |
|  | Pre-existing hypertensive chronic kidney disease complicating childbirth | O10.22 |
|  | Pre-existing hypertensive heart and chronic kidney disease complicating pregnancy, second trimester | O10.312 |
|  | Pre-existing hypertensive heart and chronic kidney disease complicating pregnancy, third trimester | O10.313 |
|  | Pre-existing secondary hypertension complicating pregnancy, first trimester | O10.411 |
|  | Pre-existing secondary hypertension complicating pregnancy, second trimester | O10.412 |
|  | Pre-existing secondary hypertension complicating pregnancy, third trimester | O10.413 |
|  | Pre-existing secondary hypertension complicating pregnancy, unspecified trimester | O10.419 |
|  | Pre-existing secondary hypertension complicating the puerperium | O10.43 |
|  | Unspecified pre-existing hypertension complicating pregnancy, first trimester | O10.911 |
|  | Unspecified pre-existing hypertension complicating pregnancy, second trimester | O10.912 |
|  | Unspecified pre-existing hypertension complicating pregnancy, third trimester | O10.913 |
|  | Unspecified pre-existing hypertension complicating pregnancy, unspecified trimester | O10.919 |
|  | Unspecified pre-existing hypertension complicating childbirth | O10.92 |
|  | Unspecified pre-existing hypertension complicating the puerperium | O10.93 |
| HELLP syndrome | HELLP syndrome (HELLP), unspecified trimester | O14.20 |
|  | HELLP syndrome (HELLP), second trimester | O14.22 |
|  | HELLP syndrome (HELLP), third trimester | O14.23 |
|  | HELLP syndrome, complicating childbirth | O14.24 |
|  | HELLP syndrome, complicating the puerperium | O14.25 |
| Unspecified maternal hypertension | Unspecified maternal hypertension, first trimester | O16.1 |
|  | Unspecified maternal hypertension, second trimester | O16.2 |
|  | Unspecified maternal hypertension, third trimester | O16.3 |
|  | Unspecified maternal hypertension, complicating childbirth | O16.4 |
|  | Unspecified maternal hypertension, complicating the puerperium | O16.5 |
|  | Unspecified maternal hypertension, unspecified trimester | O16.9 |
| Transient hypertension of pregnancy with delivery | Transient hypertension of pregnancy, with delivery, with current postpartum complication | O13.5 |
|  | Gestatnl htn without significant protein comp the puerp | O13.5 |
|  | Gestational htn w/o significant proteinuria unsp trimester OR Gestational [pregnancy-induced] hypertension without significant proteinuria, unspecified trimester | O13.9 |
|  | Transient hypertension of pregnancy, with delivery | O13.9 |
| Gestational hypertension without proteinuria | Gestational (pregnancy-induced) hypertension without significant proteinuria, first trimester | O13.1 |
|  | Gestational (pregnancy-induced) hypertension without significant proteinuria, second trimester | O13.2 |
|  | Gestational (pregnancy-induced) hypertension without significant proteinuria, third trimester | O13.3 |
|  | Gestational (pregnancy-induced) hypertension without significant proteinuria, complicating childbirth | O13.4 |
| All diagnoses were collapsed into the overall category “Maternal Hypertension”  Abbreviations: ICD-10-CM= International Classification of Diseases, 10^th^ Revision, Clinical Modification; PE=preeclampsia; HTN=hypertension; HELLP: hemolysis, elevated liver enzymes, and low platelets; w/o=without | | |
