# Supplementary material for: The relationship between maternal glucose concentrations, gestational diabetes mellitus, placental weight, and placental vascular malperfusion lesions: A retrospective study of a U.S. pregnancy cohort
Source: PLoS One. 2026 Mar 3;21(3):e0325415. doi: 10.1371/journal.pone.0325415 (PMC12956115; doi:10.1371/journal.pone.0325415)
Supplement: S2 Table — All diagnoses were collapsed into the overall category “GDM”. Abbreviations: ICD-10-CM = International Classification of Diseases, 10th Revision, Clinical Modification; GDM = Gestational diabetes mellitus; Gestatnl diab in chldbrth ctrl by oral hypoglycemic drugs = Gestational diabetes in childbirth controlled by oral hypoglycemic drugs (DOCX) [file pone.0325415.s004.docx]

| **S2 Table. Gestational diabetes mellitus diagnosis by ICD-10-CM** | | |
| --- | --- | --- |
| **Diagnosis** | **ICD-10-CM Diagnosis** | **ICD-10-CM Code** |
| GDM, diet control | Gestational diabetes mellitus in pregnancy diet controlled | O24.410 |
|  | Gestational diabetes mellitus in childbirth diet controlled | O24.420 |
|  | Gestational diabetes mellitus in the puerperium diet controlled | O24.430 |
| GDM, insulin control | Gestational diabetes in pregnancy insulin controlled | O24.414 |
|  | Gestational diabetes mellitus in childbirth insulin controlled | O24.424 |
|  | Gestational diabetes mellitus in the puerperium insulin controlled | O24.424 |
| GDM, oral drug control | Gestational diabetes in pregnancy controlled by oral hypoglycemic drugs | O24.415 |
|  | Gestational diabetes in childbirth controlled by oral hypoglycemic drugs OR Gestatnl diab in chldbrth ctrl by oral hypoglycemic drugs | O24.425 |
| GDM, unspecified control | Gestational diabetes mellitus in pregnancy unspecified control | O24.419 |
|  | Gestational diabetes mellitus in childbirth unspecified control | O24.429 |
|  | Gestational diabetes mellitus in the puerperium unspecified control | O24.439 |
| All diagnoses were collapsed into the overall category “GDM”  Abbreviations: ICD-10-CM= International Classification of Diseases, 10^th^ Revision, Clinical Modification; GDM=Gestational diabetes mellitus; Gestatnl diab in chldbrth ctrl by oral hypoglycemic drugs=Gestational diabetes in childbirth controlled by oral hypoglycemic drugs | | |
