# Supplementary material for: The relationship between maternal glucose concentrations, gestational diabetes mellitus, placental weight, and placental vascular malperfusion lesions: A retrospective study of a U.S. pregnancy cohort
Source: PLoS One. 2026 Mar 3;21(3):e0325415. doi: 10.1371/journal.pone.0325415 (PMC12956115; doi:10.1371/journal.pone.0325415)
Supplement: S4 Table — † Diagnoses are not mutually exclusive (i.e., some women have >1 GDM diagnosis). ‡ Only from the current pregnancy, history of GDM diagnoses not included. Abbreviations: GDM = gestational diabetes mellitus. (DOCX) [file pone.0325415.s006.docx]

| **S4 Table. GDM diagnostic criteria and total frequency** | |
| --- | --- |
| **Diagnosis**^†^ | **Diagnosed with GDM, n (%)**^‡^ (total n=583 pregnancies) |
| GDM, diet control | 427 (73%) |
| GDM, insulin control | 122 (21%) |
| GDM, oral drug control | 4 (0.7%) |
| GDM, unspecified control | 401 (69%) |
| † Diagnoses are not mutually exclusive (i.e. some women have >1 GDM diagnosis)  ‡ Only from the current pregnancy, history of GDM diagnoses not included  Abbreviations: GDM=gestational diabetes mellitus | |
