# Supplementary material for: The relationship between maternal glucose concentrations, gestational diabetes mellitus, placental weight, and placental vascular malperfusion lesions: A retrospective study of a U.S. pregnancy cohort
Source: PLoS One. 2026 Mar 3;21(3):e0325415. doi: 10.1371/journal.pone.0325415 (PMC12956115; doi:10.1371/journal.pone.0325415)
Supplement: S5 Table — The units for glucose challenge tests were 10 mg/dL. Interactions by infant sex and parity were not significant for any models (Wald test ≥ 0.1) and thus not included in this table. † Poisson regression model adjusted for maternal age, race (reference = NH White), parity (reference = 0), gestational age at delivery, and infant sex (reference = Female). Abbreviations: ARR = adjusted relative risk; CI = confidence interval; GCT = glucose challenge test; GDM = gestational diabetes mellitus; RR = relative risk; SE = standard error. (DOCX) [file pone.0325415.s007.docx]

| **S5 Table. Associations between glucose groups and placental lesions (n=11,585)** | | | | | | | |
| --- | --- | --- | --- | --- | --- | --- | --- |
|  |  |  |  | **Unadjusted** | | **Adjusted** | |
| **Outcome** | **Exposure** | **Total, n** | **Outcome,  n (%)** | **RR (95% CI)** | **Robust SE** | **ARR (95% CI)**† | **Robust SE**† |
| Accelerated villous maturation | Pass GCT/no GDM | 9,018 | 1,783 (20%) | Reference | | | |
|  | Fail GCT/no GDM | 1,984 | 445 (22%) | 1.13 (1.03, 1.24) | 0.05 | 1.00 (0.91, 1.10) | 0.05 |
|  | GDM | 583 | 129 (22%) | 1.12 (0.96, 1.31) | 0.09 | 1.09 (0.94, 1.27) | 0.09 |
| Increased syncytial knots | Pass GCT/no GDM | 9,018 | 927 (10%) | Reference | | | |
|  | Fail GCT/no GDM | 1,984 | 225 (11%) | 1.10 (0.96, 1.27) | 0.08 | 1.05 (0.92, 1.21) | 0.07 |
|  | GDM | 583 | 70 (12%) | 1.17 (0.93, 1.47) | 0.14 | 1.18 (0.94, 1.48) | 0.14 |
| Delayed villous maturation | Pass GCT/no GDM | 9,018 | 615 (7%) | Reference | | | |
|  | Fail GCT/no GDM | 1,984 | 136 (6%) | 1.00 (0.84, 1.20) | 0.09 | 1.08 (0.90, 1.29) | 0.10 |
|  | GDM | 583 | 46 (8%) | 1.16 (0.87, 1.54) | 0.17 | 1.36 (1.02, 1.81) | 0.20 |
| Increased perivillous fibrin deposition | Pass GCT/no GDM | 9,018 | 1,209 (13%) | Reference | | | |
|  | Fail GCT/no GDM | 1,984 | 266 (13%) | 1.00 (0.88, 1.13) | 0.06 | 0.99 (0.87, 1.12) | 0.06 |
|  | GDM | 583 | 83 (14%) | 1.06 (0.86, 1.30) | 0.11 | 1.05 (0.85, 1.30) | 0.11 |
| The units for glucose challenge tests were 10 mg/dL  Interactions by infant sex and parity were not significant for any models (Wald test ≥ 0.1) and thus not included in this table  † Poisson regression model adjusted for maternal age, race (reference = NH White), parity (reference = 0), gestational age at delivery, and infant sex (reference = Female)  Abbreviations: ARR=adjusted relative risk; CI=confidence interval; GCT=glucose challenge test; GDM=gestational diabetes mellitus; RR=relative risk; SE=standard error | | | | | | | |
