# Supplementary material for: The relationship between maternal glucose concentrations, gestational diabetes mellitus, placental weight, and placental vascular malperfusion lesions: A retrospective study of a U.S. pregnancy cohort
Source: PLoS One. 2026 Mar 3;21(3):e0325415. doi: 10.1371/journal.pone.0325415 (PMC12956115; doi:10.1371/journal.pone.0325415)
Supplement: S6 Table — A total of 753 patients were diagnosed with maternal hypertension and were excluded. † Linear regression models were adjusted for maternal age, race and ethnicity, parity, gestational age at delivery, and fetal sex. Abbreviations: AMD = adjusted mean difference; CI = confidence interval; MD = mean difference. (DOCX) [file pone.0325415.s008.docx]

| **S6 Table. Associations between glucose challenge tests (per 10 mg/dL increase) and placental weight, a sensitivity analysis excluding patients diagnosed with maternal hypertension (n=10,832)** | | | | |
| --- | --- | --- | --- | --- |
| **Outcome** | **MD (95% CI)** | ***p* value** | **AMD (95% CI)**^†^ | ***p* value** |
| Placental weight | 1.59 (0.90, 2.29) | < 0.001 | 3.15 (2.53, 3.78) | < 0.001 |
| A total of 753 patients were diagnosed with maternal hypertension and were excluded  † Linear regression models were adjusted for maternal age, race and ethnicity, parity, gestational age at delivery, and fetal sex  Abbreviations: AMD=adjusted mean difference; CI=confidence interval; MD=mean difference | | | | |
