# Supplementary material for: The relationship between maternal glucose concentrations, gestational diabetes mellitus, placental weight, and placental vascular malperfusion lesions: A retrospective study of a U.S. pregnancy cohort
Source: PLoS One. 2026 Mar 3;21(3):e0325415. doi: 10.1371/journal.pone.0325415 (PMC12956115; doi:10.1371/journal.pone.0325415)
Supplement: S7 Table — A total of 753 patients were diagnosed with maternal hypertension and were excluded. † Poisson regression models were adjusted for maternal age, race and ethnicity, parity, gestational age at delivery, and fetal sex. Abbreviations: ARR = adjusted relative risk; CI = confidence interval; LGA = large for gestational age; RR = relative risk; SGA = small for gestational age. (DOCX) [file pone.0325415.s009.docx]

| **S7 Table. Associations between glucose challenge tests (per 10 mg/dL increase) and categorical placental lesions (n=10,832)** | | | | |
| --- | --- | --- | --- | --- |
|  | **Unadjusted** | | **Adjusted**^†^ | |
| **Outcome** | **RR (95% CI)** | **Robust SE** | **ARR (95% CI)** | **Robust SE** |
| Accelerated villous maturation | 1.02 (1.01, 1.04) | 0.01 | 1.00 (0.99, 1.02) | 0.01 |
| Increased syncytial knots | 1.02 (1.00, 1.04) | 0.01 | 1.01 (0.99, 1.03) | 0.01 |
| Delayed villous maturation | 0.99 (0.97, 1.02) | 0.01 | 1.01 (0.99, 1.03) | 0.01 |
| Increased perivillous fibrin deposition | 2.00 (0.99, 1.02) | 0.01 | 1.00 (0.98, 1.02) | 0.01 |
| A total of 753 patients were diagnosed with maternal hypertension and were excluded  † Poisson regression models were adjusted for maternal age, race and ethnicity, parity, gestational age at delivery, and fetal sex  Abbreviations: ARR=adjusted relative risk; CI=confidence interval; LGA=large for gestational age; RR=relative risk; SGA=small for gestational age | | | | |
