# Supplementary material for: The relationship between maternal glucose concentrations, gestational diabetes mellitus, placental weight, and placental vascular malperfusion lesions: A retrospective study of a U.S. pregnancy cohort
Source: PLoS One. 2026 Mar 3;21(3):e0325415. doi: 10.1371/journal.pone.0325415 (PMC12956115; doi:10.1371/journal.pone.0325415)
Supplement: S8 Table — A total of 753 patients were diagnosed with maternal hypertension and were excluded. † Linear regression model was adjusted for maternal age, race and ethnicity, parity, gestational age at delivery, and fetal sex. Abbreviations: CI = confidence intervals; GDM = gestational diabetes mellitus; GCT = glucose challenge test; NH = non-Hispanic; SD = standard deviation (DOCX) [file pone.0325415.s010.docx]

| **S8 Table. Associations between glucose groups and placental weight, a sensitivity analysis excluding patients diagnosed with maternal hypertension (n=10,832)** | | | | | |
| --- | --- | --- | --- | --- | --- |
|  |  | **Unadjusted** | | **Adjusted**^†^ | |
| **Glucose group** | **Mean (SD)** | **Mean difference (95% CI)** | ***p* value** | **Mean difference (95% CI)** | ***p* value** |
| **Placental weight (grams)** | | | | | |
| **Pass GCT/no GDM** | 434 (109) | Reference | | | |
| **Fail GCT/no GDM** | 439 (113) | 5.3 (-0.2, 10.8) | 0.061 | 12.8 (7.9, 17.7) | < 0.001 |
| **GDM** | 447 (108) | 12.9 (3.3, 22.5) | 0.009 | 21.3 (1.3, 29.9) | < 0.001 |
| A total of 753 patients were diagnosed with maternal hypertension and were excluded  † Linear regression model was adjusted for maternal age, race and ethnicity, parity, gestational age at delivery, and fetal sex  Abbreviations: CI=confidence intervals; GDM=gestational diabetes mellitus; GCT=glucose challenge test; NH=non-Hispanic; SD=standard deviation | | | | | |
