# Supplementary material for: The relationship between maternal glucose concentrations, gestational diabetes mellitus, placental weight, and placental vascular malperfusion lesions: A retrospective study of a U.S. pregnancy cohort
Source: PLoS One. 2026 Mar 3;21(3):e0325415. doi: 10.1371/journal.pone.0325415 (PMC12956115; doi:10.1371/journal.pone.0325415)
Supplement: S9 Table — A total of 753 patients were diagnosed with maternal hypertension and were excluded. Poisson regression model was adjusted for maternal age, race and ethnicity, parity, gestational age at delivery, and fetal sex Abbreviations: CI = confidence intervals; GDM = gestational diabetes mellitus; GCT = glucose challenge test (DOCX) [file pone.0325415.s011.docx]

| **S9 Table. Associations between glucose groups and categorical outcomes, a sensitivity analysis excluding patients diagnosed with maternal hypertension (n=10,832)** | | | | | | | |
| --- | --- | --- | --- | --- | --- | --- | --- |
|  |  |  |  | **Unadjusted** | | **Adjusted** | |
| **Outcome** | **Exposure** | **Total, n** | **Outcome, n (%)** | **RR (95% CI)** | **Robust SE** | **ARR (95% CI)**^†^ | **Robust SE** |
| Accelerated villous maturation | Pass GCT/no GDM | 8,457 | 1,568 (19%) | Reference | | | |
|  | Fail GCT/no GDM | 1,847 | 387 (21%) | 1.13 (1.02, 1.25) | 0.06 | 1.00 (0.90, 1.11) | 0.05 |
|  | GDM | 528 | 111 (21%) | 1.13 (0.06, 1.35) | 0.10 | 1.10 (0.93, 1.30) | 0.01 |
| Increased syncytial knots | Pass GCT/no GDM | 8,457 | 849 (10%) | Reference | | | |
|  | Fail GCT/no GDM | 1,847 | 213 (12%) | 1.15 (0.10, 1.32) | 0.08 | 1.09 (0.95, 1.26) | 0.08 |
|  | GDM | 528 | 62 (12%) | 1.17 (0.92, 1.49) | 0.14 | 1.17 (0.92, 1.49) | 0.14 |
| Delayed villous maturation | Pass GCT/no GDM | 8,457 | 595 (7%) | Reference | | | |
|  | Fail GCT/no GDM | 1,847 | 132 (7%) | 1.02 (0.85, 1.22) | 0.09 | 1.08 (0.10, 1.30) | 0.01 |
|  | GDM | 528 | 40 (8%) | 1.08 (0.79, 1.46) | 0.17 | 1.27 (0.93, 1.72) | 0.20 |
| Increased perivillous fibrin deposition | Pass GCT/no GDM | 8,457 | 1,118 (13%) | Reference | | | |
|  | Fail GCT/no GDM | 1,847 | 247 (13%) | 1.01 (0.89, 1.15) | 0.07 | 1.00 (0.88, 1.14) | 0.07 |
|  | GDM | 528 | 74 (14%) | 1.06 (0.85, 1.32) | 0.12 | 1.05 (0.84, 1.31) | 0.12 |
| A total of 753 patients were diagnosed with maternal hypertension and were excluded  † Poisson regression model was adjusted for maternal age, race and ethnicity, parity, gestational age at delivery, and fetal sex  Abbreviations: CI=confidence intervals; GDM=gestational diabetes mellitus; GCT=glucose challenge test | | | | | | | |
